# Prospects of microbial cell factories developed through systems metabolic engineering

**DOI:** 10.1111/1751-7915.12385

**Published:** 2016-07-20

**Authors:** Martin Gustavsson, Sang Yup Lee

**Affiliations:** ^1^ Metabolic and Biomolecular Engineering National Research LaboratoryDepartment of Chemical and Biomolecular Engineering (BK21 Plus Program)BioProcess Engineering Research CenterCenter for Systems and Synthetic BiotechnologyInstitute for the BioCenturyKorea Advanced Institute of Science and Technology (KAIST)291 Daehak‐ro, Yuseong‐guDaejeon34141Korea; ^2^ KTH Royal Institute of TechnologySchool of BiotechnologyDivision of Industrial BiotechnologyAlbaNova University Center106 91StockholmSweden

## Abstract

While academic‐level studies on metabolic engineering of microorganisms for production of chemicals and fuels are ever growing, a significantly lower number of such production processes have reached commercial‐scale. In this work, we review the challenges associated with moving from laboratory‐scale demonstration of microbial chemical or fuel production to actual commercialization, focusing on key requirements on the production organism that need to be considered during the metabolic engineering process. Metabolic engineering strategies should take into account techno‐economic factors such as the choice of feedstock, the product yield, productivity and titre, and the cost effectiveness of midstream and downstream processes. Also, it is important to develop an industrial strain through metabolic engineering for pathway construction and flux optimization together with increasing tolerance to products and inhibitors present in the feedstock, and ensuring genetic stability and strain robustness under actual fermentation conditions.

## Introduction

Our increasing concerns on climate change and the eventual depletion of fossil resources have directed much research towards developing renewable ways for producing chemicals and fuels. One strategy that has been under investigation for many years, beginning at the industrial‐scale with conversion of simple sugars into acetone, butanol and ethanol 100 years ago (Moon *et al*., 2016), and more heavily in recent years, is the utilization of microorganisms for producing chemicals and fuels from renewable non‐food biomass. This field has advanced tremendously over the past two decades, revolutionized by technological developments allowing inexpensive large‐scale genome sequencing, systems‐level gene expression profiling and other omics techniques, *in silico* metabolic modelling and simulation, enzyme/pathway engineering and evolution, in addition to routine and upgraded recombinant DNA techniques. Despite this, the number of examples of successful commercialization of microbial processes producing chemicals and fuels remains relatively small to date (Table 1), leaving the question of whether a large‐scale switch towards a sustainable society based on microbial biorefineries is feasible. To achieve this goal, it is critical that metabolic engineering works are designed and performed with the full techno‐economic picture in mind, considering the raw material availability, large‐scale production and downstream processes, and even applications throughout the strain development. To reach this goal, interactions between academia and industry are obviously vital (Pronk *et al*., 2015). In this work, we review the techno‐economic challenges for the large‐scale implementation of biorefineries with the producing microbial cell factories as the focus, and discuss metabolic engineering strategies to overcome the pending challenges. We have recently suggested 10 general and stepwise strategies to consider for strain development suitable for industrial biorefineries (Lee and Kim, 2015); thus, here we rather focused on complementary points to be considered.

**Table 1:** Status of commercialization of microbial cell factories

Product	Production organism	Status	Feed stock	Companies	Reference
Chemicals					
Acetone	*Clostridium acetobutylicum*	Commercialized	Corn	Green Biologics	www.greenbiologics.com
Citric acid	*Aspergillus niger*	Commercialized			
Succinic acid	*E. coli*	Commercialized	Corn sugars	BioAmber	www.bio-amber.com
	*E. coli*	Commercialized	Sucrose	Myriant	www.myriant.com
	*S. cerevisiae*	Commercialized	Starch, sugars	Reverdia	www.reverdia.com
	*B. succiniproducens*	Commercialized	Glycerol, sugars	Succinity	www.succinity.com
Lactic acid		Commercialized	Corn sugars + more	NatureWorks	www.natureworksllc.com
Itaconic acid	*Aspergillus terreus*	Commercialized	Biochemistry	Qingdao Kehai	www.kehai.info/en
1,3‐PDO	*E. coli*	Commercialized	Corn sugars	DuPont Tate & Lyle	www.duponttateandlyle.com
1,3‐BDO		Demonstration		Genomatica and Versalis	www.genomatica.com
1,4‐BDO	*E. coli*	Commercialized	Sugar	Genomatica and DuPont Tate & Lyle	www.genomatica.com
1,5‐PDA		Commercialized	Sugar	Cathay Industrial Biotech	www.cathaybiotech.com
3‐HP		Commercialized		Metabolix	www.metabolix.com
		Demonstration		Novozymes and Cargill	www.novozymes.com
Isoprene	*S. cerevisiae*	Preparing	Sugar, cellulose	Amyris, Braskem, Michelin	www.amyris.com
		Preparing		DuPont, Goodyear	www.biosciences.dupont.com
Isobutene	*E. coli*	Demonstration	Glucose, sucrose	Global Bioenergies	www.global-bioenergies.com
Adipic acid	*Candida* sp.	Demonstration	Plant oils	Verdezyne	www.verdezyne.com
Sebacic acid	*Candida* sp.	Demonstration	Plant oils	Verdezyne	www.verdezyne.com
DDDA	*Candida* sp.	Under commercialization	Plant oils	Verdezyne	www.verdezyne.com
Squalene	*S. cerevisiae*	Commercialized	Sugarcane	Amyris	www.amyris.com
PHA	*E. coli*	Commercialized		Metabolix	www.metabolix.com
Fuels					
Ethanol	*S. cerevisiae*,* Zymomonas mobilis*,* Kluyveromyces marxianus*	Commercialized	Sugarcane, corn sugar, lignocellulose	Many	
	*Clostridium autoethanogenum*	Demonstration	Flue gas	Lanzatech	www.lanzatech.com
Farnesene	*S. cerevisiae*	Commercialized		Amyris	www.amyris.com
Butanol	*Clostridium acetobutylicum*	Commercialized	Corn	Green Biologics	www.greenbiologics.com
Isobutanol	Yeast	Commercialized	Sugars	Gevo	www.gevo.com

1,3‐PDO, 1,3‐propanediol; 1,3‐BDO, 1,3‐butanediol; 1,4‐BDO, 1,4‐butanediol; 1,5‐PDA, 1,5‐pentanediamine; 3‐HP, 3‐hydroxypropionic acid; DDDA, dodecanedioic acid; PHA, polyhydroxyalkanoates.

## Techno‐economic considerations for microbial cell factory development

**Figure 1:**
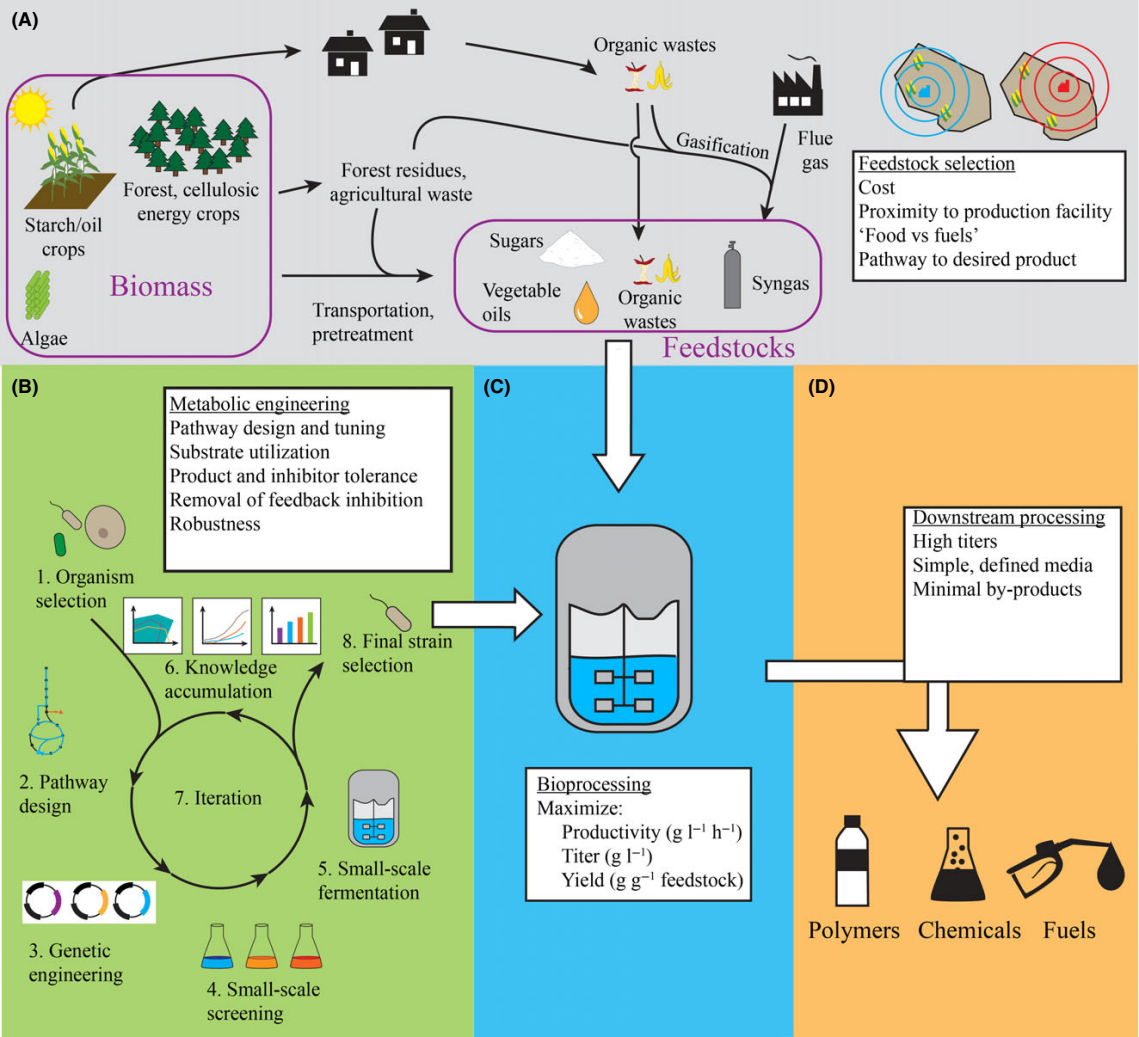
Overview of the microbial cell factory design process. For successful commercial implementation, the full picture should be considered throughout the design process. A. Considerations relating to the choice of renewable feedstock, including the location of the production facility in close proximity to the available feedstocks, as indicated by blue (correct) and red (incorrect) concentric circles. B. The metabolic engineering process, starting from selection of production organisms and iterating through design‐build‐test‐learn cycles until the process requirements are met. C and D. Critical parameters for the production process and downstream purification to final products respectively.

To achieve the successful transition from laboratory‐scale demonstration to large‐scale commercial production, there are three key performance indicators to consider (Fig. 1): the product yield (g g^−1^ substrate), the productivity (g l^−1^ h^−1^) and the product titre (g l^−1^). In the case of bulk chemicals and fuels, the profit margins are very narrow and it is critical that these three metrics are maximized to be competitive with traditional, petrochemical processes. In many (but not all) cases, a trade‐off has to be made between productivity and yield, as maximizing the productivity often requires high density of the producing microbes, while increasing the carbon flux directed towards cell mass inevitably lowers product yield. In an industrial‐scale fermentation, often performed in a fed‐batch mode, a productivity of typically around 2–5 g l^−1^ h^−1^ (and preferably higher) needs to be achieved depending on the product. Another strategy for increasing productivity is the use of continuous cultivation, but as these are prone to costly contamination and phage infection, the process requires more expensive equipment, and places high demand of genetic stability on the production host (Croughan *et al*., 2015). The fed‐batch cultivation strategy should be developed based on the product formation characteristics, e.g. growth associated or non‐growth associated, and strains should be further optimized to meet the process requirements. For the production of bulk and inexpensive products, the use of rather expensive inducers is discouraged; consequently, constitutive expression of metabolic genes is often employed.

The downstream processes for the separation and purification of a product should also be considered already at the strain development stage (Fig. 1). The final product titre has a major impact on the downstream processing costs, as a highly concentrated product is both easier to separate and allows reduction of the total volume to be handled (including during waste treatment). The use of a chemically defined medium not only allows production of a desired product under more controllable conditions but also simplifies the downstream purification by limiting the amount of contaminants. For the same reason, minimizing by‐product formation is essential during strain development to lower the downstream processing costs. The downstream processes can also be simplified by altering the fermentation conditions from typical ones together with the matching strain developed. For example, in the industrial production of carboxylic acids, it is advantageous to perform the fermentation process at a low pH as this leads to direct precipitation of the protonated acid (the desired product) rather than its salt form. This is why Cargill and Reverdia use acid‐tolerant yeast strains to produce lactic and succinic acids respectively, at low pH (Table 1).

The feedstock is another major contributor to the total bioprocess costs, especially for large volume low price products; for instance, the feedstock cost is estimated to be around 75% of the total production cost of U.S. corn‐based ethanol (FAO, 2008). Thus, selecting an appropriate feedstock is of outmost importance for cheap bulk products. So far, most commercial processes have focused on using simple sugars from sugar cane and starch (Table 1). However, a switch towards utilizing non‐edible lignocellulosic biomass is highly desirable in order to avoid conflicts with food uses. Lignocellulose is the most abundant biomass on earth with an estimated annual production of around 150–170 × 10^9^ tons, out of which only around 2 × 10^9^ tons are currently used for energy, timber, and paper and pulp production (Pauly and Keegstra, 2008). Nevertheless, the necessity for extensive pre‐treatment together with the presence of microbial growth inhibitors, make these feedstocks more difficult to work with compared with sugarcane sugar or starch (Jönsson *et al*., 2013). In fact, sugarcane is an excellent feedstock, due to its relatively low cost of production, high sugar yield and low energy input requirements for its cultivation (Renouf *et al*., 2008). However, it is abundantly available only in several specific regions including Brazil, Australia and Southeast Asian countries. This highlights a major consideration for feedstock selection: proximity to the chosen substrates, as shown in Fig. 1A. The costs of feedstock transportation contribute considerably to the costs of raw materials; estimated at up to 30% for locally sourced lignocellulosic feedstocks (Hess *et al*., 2007). Thus, it makes a lot of sense to base a Brazilian biorefinery on sugarcane, while corn has been the focus of US bioethanol production efforts (EIA, 2016). Northern countries with less favourable agricultural climates should carefully calculate the transportation costs for the use of these feedstocks, or instead focus on other feedstocks, such as the abundant forest industry residues available in Canada, Finland and Sweden.

In addition to using biomass directly as a feedstock, there have been investigations into feeding CO_2_ or gasified biomass in the form of synthesis gas (syngas) into biorefineries, with the former strategy bypassing the need to first fix CO_2_ into plant biomass. Direct CO_2_ utilization has been investigated using photosynthetic microalgae or cyanobacteria, either to produce oils for further conversion or for direct production of target chemicals and fuels. This technology promises superior oil yields per cultivated area compared with conventional oil crops, but much work still remains to bring costs down to levels comparable to common vegetable oils (Chisti, 2007; Hannon *et al*., 2010). As for syngas, it can be obtained by gasification of various biomass components or municipal organic wastes using thermochemical processes (Puigjaner, 2011). The resulting gas can in turn be fermented by acetogens such as *Clostridium ljungdahlii* or *Clostridium carboxidivorans*. These bacteria convert CO and CO_2_ into fuels or chemicals, including acetate, methane, ethanol and acetone, using hydrogen as a source for reducing power through the Wood–Ljungdahl pathway (Molitor *et al*., 2016). There is much ongoing effort on strain and bioprocess development using one‐carbon substrates for the production of chemicals and fuels, as exemplified by Lanzatech's ongoing commercialization of bioconversion of industrial off‐gases (Table 1).

## Systems metabolic engineering for developing microbial cell factories

The key role of metabolic engineers in the creation of commercial microbial cell factories is engineering strains that can produce the target product efficient enough to meet the requirements for large‐scale bioprocessing as discussed above and summarized in Fig. 1. These requirements include utilization of a wide range of carbon sources, for instance the common 5‐ and 6‐carbon monosaccharides including glucose, xylose and arabinose present in lignocellulose, disaccharides including sucrose and lactose, and fatty acids, depending on feedstock availability. Furthermore, the organism should be tolerant to inhibitors present in these feedstocks and high titres of the target product, allow easy process design by growing fast in a simple defined medium, be robust in relation to heterogeneities in substrate, pH, temperature and oxygen levels typically found in large‐scale bioprocesses, be genetically stable over many divisions (at least 30 generations when scaling from 100 ml to 10 000 m^3^ production scale) and insensitive to infections by for instance bacteriophages. Finally, the production host should obviously possess metabolic capacities for production of the target product at high productivities and yields.

### Starting strain selection

The metabolic engineering process begins with the selection of the base strain for modification. As it is unlikely that a single wild‐type organism will have a phenotype covering all the requirements for the production of diverse products, the selection of a starting strain considering metabolic capacity towards the desired product, bioprocess compatibility, easiness of metabolic and genetic engineering, ability to utilize inexpensive feedstocks and others. So far, many studies have focused on engineering simple model organisms, including *Escherichia coli* and *S. cerevisiae*, as these microorganisms have been relatively more studied, have well‐developed tools for various genetic manipulations and have well‐validated genome‐scale metabolic models available for genome‐scale metabolic simulations. Several other strains that have also been employed for biorefineries include *Corynebacterium glutamicum Clostridium* sp., *Bacillus* sp. and *Pseudomonas* sp. Nevertheless, with recent developments in inexpensive genome sequencing, more rapid construction of genome‐scale metabolic models, new genetic and genomic manipulation tools, most notably the use of clustered regularly interspersed short palindromic repeats (CRISPR) for metabolic engineering of microbes (Li *et al*., 2015; Tong *et al*., 2015; Blin *et al*., 2016), metabolic engineering of microorganisms including non‐conventional host strains has become easier than in the past.

One good example of using a non‐model organism to achieve efficient chemical production is the metabolic engineering of *Mannheimia succiniproducens* for succinic acid production. This organism was isolated from the rumen of a Korean cow, an environment with a high CO_2_ partial pressure. The reason for screening the cow rumen was based on the hypothesis that there might be a bacterium capable of efficiently performing phosphoenolpyruvate carboxylation in this CO_2_‐rich environment, which was indeed the case. After genome sequencing and generation of a validated genome‐scale model, development of expression vectors and genetic manipulation tools, and systems metabolic engineering, a final strain capable of producing succinic acid at titres, yields and productivities comparable to or exceeding those of the best current industrial producers could be developed (Choi *et al*., 2016b).

*In silico* genome‐scale metabolic modelling and simulation can be a great tool in selecting an appropriate production organism, by allowing evaluation of the metabolic capacities of different organisms. One example is a recent extensive evaluation of the *E. coli* capacities for biosynthesis of a large range of chemicals (Zhang *et al*., 2016). This study found that up to 1777 non‐native products, out of which 279 have known commercial applications, could be derived from the *E. coli* metabolism by introducing heterologous enzymes.

### Pathway design for novel products

One of the greatest challenges faced by metabolic engineers is the creation of pathways for target products with no known natural producer. For these products, new enzymes need to be developed, starting from ones catalysing similar reactions as the desired one. Identification of such suitable enzyme candidates is no easy task, but recent developments in computer tools are very helpful for this. For instance, the pathway for 1,4‐butanediol production in *E. coli* could efficiently designed and tested by using the SimPheny Biopathway Predictor (www.genomatica.com; Yim *et al*., 2011). A number of such tools exist for prediction of pathways and selection of enzyme candidates, as reviewed elsewhere (Shin *et al*., 2013). Additionally, cell‐free pathway assembly tools more recently reported promises rapid screening of heterologous pathways *in vitro* (Karim and Jewett, 2016).

### Removing negative regulatory circuits

When overproducing some natural metabolites, a common problem encountered is feedback inhibition and transcriptional attenuation control of the production pathway caused by accumulation of the desired product. Such negative regulations can occur both at the transcriptional level and be caused by allosteric regulation of pathway enzymes, and should be removed at the early stage of strain development. In the case of transcriptional regulation, the strategy is straightforward. Modern DNA‐manipulation tools allow relatively simple editing of chromosomal transcription regions to introduce desired changes. Alternatively, the transcription factors involved in this regulation may also be knocked out. Examples of these strategies include the production of different amino acids (pathways that are heavily affected by such negative regulatory circuits); a number of examples are available, for instance knocking out the aromatic amino acid biosynthesis pathway regulator tyrosine‐activated repressor (t*yrR*) to allow production of L‐tyrosine (Lütke‐Eversloh and Stephanopoulos, 2007) and its derivative phenol (Kim *et al*., 2014), and replacing the native promoters of the L‐valine and L‐threonine operons with a strong, inducible promoter for more efficient production of L‐valine and L‐threonine, respectively, in *E. coli* (Lee *et al*., 2007; Park *et al*., 2007).

Dealing with allosteric feedback inhibition is more difficult compared with transcriptional regulation. The most straightforward solution is to look for heterologous enzymes that lack the allosteric regulation. Alternatively, enzyme engineering can be employed to generate feedback‐resistant mutants. This was exemplified for production of L‐threonine using *E. coli*, where previously identified single point mutations in the *thrA* and *lysC* genes (encoding aspartokinase I and III respectively) were used to remove allosteric feedback regulation of this pathway (Lee *et al*., 2007), and for the production of L‐tyrosine, where a single mutation removed the allosteric regulation of *aroG* and two mutations removed the regulation of *tyrA* (Lütke‐Eversloh and Stephanopoulos, 2007; Kim *et al*., 2014).

### Fine‐tuning pathway expression and minimizing metabolic burden

After establishment of a deregulated production pathway to the target product, the next step is fine‐tuning of the pathway to maximize the flux to the target product while minimizing the metabolic burden. As the number of pathway enzymes increase, the task of tuning the expression of each one quickly becomes very large, as shown in a recent work describing optimization of violacein production in *E. coli* (Jones *et al*., 2015). In this work, a five‐step pathway for violacein production was cloned combinatorially under five different promoter sequences, resulting in a randomized library of 3125 variants. By screening a fraction of this library, a strain with a 62‐fold improvement in violacein production over a control having only strong promoter variants was obtained.

As an alternative to promoter engineering, the influence of the Shine–Dalgarno sequence was systematically investigated in *E. coli*. A tool named Empirical Modeling and Oligos for Protein Expression Changes (EMOPEC, http://emopec.biosustain.dtu.dk) was developed for generating oligo sequences for tuning the translation of chromosomal genes in *E. coli* (Bonde *et al*., 2016). This tool promises efficient generation of combinatorial libraries for high‐throughput tuning of chromosomal pathways. Another recent contribution of interest describes the use of a chromosomally integrated green fluorescent protein (GFP) under a constitutive promoter as a reporter for examining the protein synthesis capacity of an engineered cell (Ceroni *et al*., 2015). By comparing the GFP expression in strains containing different plasmid constructs, they were able to select for constructs that produced the optimum level of the target protein, while minimizing the reduction in capacity for synthesis of other proteins. Combining this method with the above‐mentioned combinatorial library techniques could greatly assist in future pathway‐tuning efforts.

Furthermore, inducer‐free expression systems are preferable in industry to remove the cost of the inducer. Traditionally, this has been achieved using constitutive promoters, but this prevents separation of cell growth and product formation. It is envisioned that the progress in design of synthetic circuits, not least the recent report of a software for automatic design of biological circuits with predictable output (Nielsen *et al*., 2016), will lead to the development of advanced, dynamically regulated auto‐induction strategies in the future.

### Substrate utilization engineering

Not only should the production organism contain efficient, well‐tuned production pathways as discussed above but it also needs to efficiently utilize the inexpensive feedstock of choice. A classic example of this problem is that *S. cerevisiae* is incapable of growing on xylose (Jeffries and Jin, 2004). As xylose typically makes up around 15–23% of carbohydrates in lignocellulose (Huang *et al*., 2009), this is a critical issue that needs to be solved to obtain higher yields from this feedstock. Consequently, this was one of the most heavily studied topics, and there are now a number of reports describing engineered *S. cerevisiae* for this purpose, as reviewed elsewhere (Laluce *et al*., 2012). Other organisms, including for instance *E. coli*, can natively utilize the five‐carbon carbohydrates arabinose and xylose. However, in *E. coli* a strict substrate utilization hierarchy is maintained, with a preference for glucose utilization before other carbohydrates. In order for efficient fed‐batch processing of mixed lignocellulosic sugars, this hierarchy needs to be removed to enable simultaneous sugar utilization. One such example is the deletion of the *E. coli* glucose phosphotransferase system domain IIC (encoded by *ptsG*) that removes this substrate preference from *E. coli*. This deletion was recently used to enable synthesis of poly(lactate‐co‐glycolate) through simultaneous utilization of glucose and xylose (Choi *et al*., 2016a). Another example is the inability of the commonly used *E. coli* K12 and B strains to utilize sucrose as a carbon source; simple overexpression of a single β‐fructofuranosidase gene from *M. succiniproducens* could confer the sucrose‐utilization phenotype to an engineered *E. coli* K12 strain, allowing direct production of L‐threonine from sucrose (Lee *et al*., 2010).

### Engineering cells to tolerate target products and inhibitors present in feedstock

For utilization of complex substrates such as lignocellulosic biomass and accumulation of target products to high titres, it is necessary that industrial strains should be resistant towards both the target product and inhibitors present in the feedstock. However, inhibitor tolerance is one of the most complex phenotypes to engineer as a wide number of hard‐to‐predict genes are often involved. Thus, this phenotype is commonly achieved by one of two main strategies. First, adaptive laboratory evolution can be used, as described for production of D‐lactate in *E. coli* (Utrilla *et al*., 2012) and L‐arginine in *C. glutamicum* (Park *et al*., 2014). Following the genome sequencing of evolved strains, the contributing alterations from adaptive laboratory evolution can then be introduced into the production strain. Second, system‐wide genomic or transcriptomic engineering followed by screening and selection can also be used. For this, various methods including transcription factor engineering (Alper *et al*., 2006), and gene knockdown libraries based on sRNA (Na *et al*., 2013) or CRISPR interference (CRISPRi; Qi *et al*., 2013) can be employed.

A different, rational engineering strategy to improve both productivity and product tolerance is to express product efflux pumps that enhance the export of the product. One example of this strategy is the screening of a library of 43 efflux pumps for improving the tolerance of *E. coli* against seven different biofuels, resulting in the identification of efflux pumps improving the tolerance against five of seven of the tested biofuels (Dunlop *et al*., 2011). Another recent work describes the screening of 16 ABC transporters for the ability to enhance secretion of carotenoids from *E. coli* (Doshi *et al*., 2013). Using this strategy, up to 5.4‐fold increase in carotenoid secretion was achieved while maintaining cell viability.

### Genetic stability and strain robustness

Industrial strains need to be stable over a great number of generations to allow scaling up to production scale. This is no small challenge, as cost issues and environmental concerns prohibit the use of antibiotics at production scale. Thus, it is preferable to use chromosomal pathway integration over plasmid‐based expression if possible. The invention of the CRISPR system has greatly simplified this, as exemplified by a recent report describing the integration of a five‐gene pathway in a single step using CRISPR (Bassalo *et al*., 2016). Furthermore, pathway tuning to minimize the metabolic burden of the production pathway is necessary to avoid preferential selection of non‐producers. To combat this, biosensors are developed to couple target products to an essential gene for cell growth (Xiao *et al*., 2016). In this design, cells with a high production rate obtain a growth advantage compared with low‐producers, leading to the highest fatty acids titre reported to date in *E. coli* (21.5 g l^−1^). Such approaches will be more frequently employed as synthetic biology research advances rapidly.

Finally, the production host must also be robust towards the stresses experienced in large‐scale bioprocesses, such as inhomogeneities resulting from insufficient mixing. This is why pilot plant testing (and even domo‐plant testing) is important. It has also been suggested to repeatedly test strains under process‐like conditions during the strain development process, for instance using scale‐down bioreactor systems (Neubauer and Junne, 2016). Biotechnology companies have accumulated much unpublished knowledge on scaling‐up process, and thus academia‐industry collaboration will speed up the successful scale‐up process.

## Conclusion

In this work, we have briefly reviewed the challenges associated with the establishing industrial‐scale microbial biorefineries for the production of chemicals and fuels. As has been discussed, many different aspects need to be taken into account during the strain development by metabolic engineering. The most important lesson for successful cell factory development is thus to have an all‐encompassing view during strain development, including everything from feedstock selection, through the bioprocessing steps and to the downstream processes. It is clear that such rapid advances in the field promise successful establishment of microbial biorefineries producing chemicals and fuels from renewable non‐food biomass, allowing us to move towards a bio‐based economy.

## Conflict of interest

The authors declare no conflicts of interest.
